# Ethanol production from lignocellulosic waste materials: kinetics and optimization studies

**DOI:** 10.1039/d5ra02272j

**Published:** 2025-07-22

**Authors:** Naeemah A. Ibrahim, Halah Hameed Majeed, Rand A. Abid, G. Abdulkareem Alsultan, N. Asikin Mijan, H. V. Lee, Tonni Agustiono Kurniawan, Yun Hin Taufiq-Yap

**Affiliations:** a Southern Technical University Basra Iraq; b Catalysis Science and Technology Research Centre, Faculty of Science, Universiti Putra Malaysia 43400 Serdang Selangor Malaysia taufiq@upm.edu.my abdulkareem@upm.edu.my; c Department of Chemical Sciences, Faculty of Science and Technology, Universiti Kebangsaan Malaysia 43600 UKM Bangi Selangor Darul Ehsan Malaysia; d Nanotechnology and Catalysis Research Centre (NanoCat), Institute of Postgraduate Studies, University Malaya 50603 Kuala Lumpur Malaysia leehweivoon@um.edu.my; e College of Environment and Ecology, Xiamen University Xiamen 361102 China

## Abstract

This study investigates the composition, hydrolysis, fermentation, kinetic studies and optimization by response surface methodology (RSM) of ten different lignocellulosic materials in ethanol production using enzymatic hydrolysis of isolated *Trichoderma reesei and Aspergillus niger* and fermentation by *Zymomonas mobilis* and *Saccharomyces cerevisiae*. Proximate and ultimate analyses reveal that sugarcane bagasse and rice husk are ideal feedstocks due to their high volatile matter, low moisture, and ash content, offering more fermentable carbohydrates. The highest glucose concentrations were achieved from sugarcane bagasse (0.5689 g L^−1^) using *T. reesei* and from rice husk (0.5803 g L^−1^) using *A. niger*. Pretreatment increased glucose yields, with rice husk (RHAn) yielding 9.3 g L^−1^ ethanol in 60 h and sugarcane bagasse (SBTr) yielding 8.1 g L^−1^ in 48 h, and the particle size reduction to 75 μm enhanced glucose yields due to increased surface area. Kinetic models, including the Monod and Michaelis–Menten models, were used to describe ethanol production, with RHAn exhibiting the highest growth parameters. This study reports optimized ethanol production that achieved maximum yields under controlled conditions, further supporting the feasibility of large-scale bioethanol production.

## Abstract 2

This study explores the composition, hydrolysis, fermentation, kinetics, and RSM-based optimization of ten lignocellulosic materials for ethanol production using enzymes from *T. reesei*, *A. niger*, and fermentation by *Z. mobilis* and *S. cerevisiae*.

## Graphical abstract

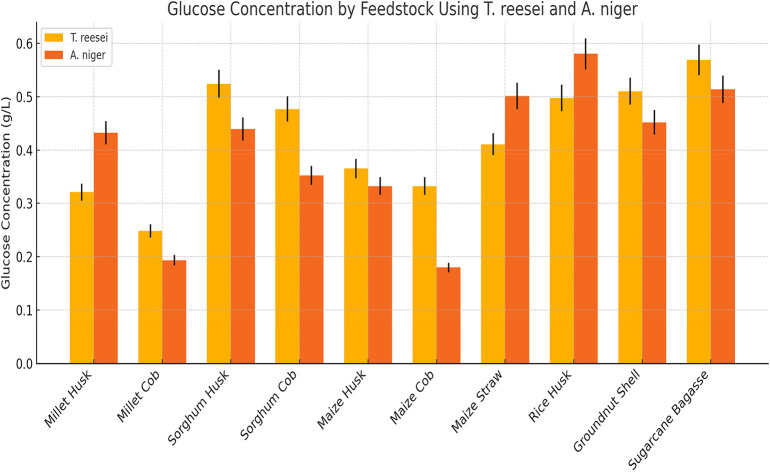

## Introduction

Due to the worldwide overreliance on fossil fuel-based energy and increasing concerns about the environment, there is a global interest in developing alternative fuel and energy sources that are renewable and sustainable.^1,2^ Some of the physical effects of climate change that have already occurred include sea level rise, melting glaciers and polar ice, and increased risks of heatwaves, floods, and droughts. Therefore, a rapid and significant reduction in greenhouse gas emissions is required to prevent these effects from worsening and threatening the survival of most life on Earth.^3,4^

Two-thirds (66%) of the world's energy consumption comes from conventional nonrenewable fuels, including coal (27%), petrol (34%), and oil (24%). This has led to significant price increases and substantial environmental risks such as climate change and global warming.^5^ Renewable energy sources, such as biomass, solar, wind, hydropower, biodiesel and geothermal energy,^2,6^ have emerged as competitive substitutes for fossil fuels. Over the last three decades, 56% of research studies on renewable energy have focused on biomass, which is considered an ideal substitute for fossil fuels as an energy source.^7–9^

With an annual global production of over 182 billion tonnes, of which only about 8 billion tonnes are currently utilised, lignocellulosic biomass is the most common renewable raw resource.^10^ The renewable energy generated from agricultural biomasses has the potential to substitute fossil fuel generation.^11^ Agricultural waste can be safely and affordably disposed of *via* bioconversion, which also has the ability to turn lignocellulosic wastes into useful forms like reducing sugars for ethanol production.^12–14^ About 3–4% of all ethanol produced worldwide is produced synthetically. The remainder is produced through the fermentation of biomass, comprising mostly cereals and sugar crops such as cane and beetroot.^15,16^

Lignocellulosic biomass comprises about 30% to 50% cellulose, 15 to 30 weight percent (wt) of hemicellulose, and 10 to 30 weight percent (wt) of lignin, as well as smaller amounts of (organic and inorganic) extractives and other inorganic compounds, depending on the type of plant.^17,18^ Cellulosic ethanol, another name for second-generation (2 G) ethanol, is generated from lignocellulosic biomass.^19^ The primary sources of biomass are forestry wastes, crops, animal and industrial residues, sewage, and municipal solid waste. Biomass is mostly sourced from plants and plant-derived chemicals.^20^

The cultivation and processing of maize (corn stover), wheat (wheat straw), rice (rice straw), sorghum (sorghum stalks), barley (barley straw) and sugarcane (sugarcane bagasse) are the main sources of agro-based lignocellulosics with high cellulose and hemicellulose contents.^15,18^ Therefore, it is possible to establish non-food biomass feedstock for the environmentally-friendly, sustainable manufacturing of transportation fuels from biomass resources. The three primary steps of the bioconversion process are pretreatment, hydrolysis, and the fermentation process.^21^ Kinetic models are required for chemical and biochemical processes because they describe the process performance. For example, enzymatic hydrolysis kinetic models can be used to understand how an enzyme interacts with its substrate in the generation of ethanol from lignocellulose materials. A few investigations studied the kinetics and optimization of the process variables of ethanol production from lignocellulose materials. For example, to maximises the hydrolysis and fermentation of *Colocynthis vulgaris* Shrad seed shell (CVSSS), the Box–Behnken Design (BBD) of Response Surface Methodology (RSM) was used and a kinetic study was conducted. However, their investigation was restricted to a single culture (*Aspergillus niger*). With a yield of 0.38 g ethanol per g substrate, volumetric productivity of 0.64 g L^−1^ h^−1^, and fermentation efficiency of 73.6%, rice straw (RS) grown with *Candida tropicalis* achieved the highest ethanol concentration of 15.3 g L^−1^ in a 24 hours period.^22,23^

In a previous study,^24^ the operating conditions of the xylanase production process were optimised (medium pH and incubation temperature). Bioethanol was synthesised from lignocellulosic biomass using xylanase. Other hydrolytic enzymes (*Aspergillus niger*) produce xylanase under submerged fermentation when oil palm empty fruit brunches were used as carbon sources. In another study, the validity of the kinetic model was tested using three different agri-food residues: rice husks, wheat straw, and exhausted sugar beetroot pulp.^25^ It was discovered that some crucial operating variables, like the enzyme dose and the inoculum strength, must be well coupled to obtain the maximum yield from residues. Two kinetic models were combined to create a general kinetic model for the simultaneous saccharification and fermentation of lignocellulosic materials.^26,27^

The objectives of this study are to carry out the kinetic studies of enzymatic hydrolysis and fermentation, and to optimize the ethanol production from lignocellulose materials using a mixed culture of isolated *Aspergillus niger*, *Trichoderma reesei*, (enzymatic hydrolysis) and *Saccharomyces cerevisiae* and *Zymomonas mobilis* (fermentation). The novelty of this study lies in the synergistic use of mixed microbial cultures for efficient ethanol production from lignocellulosic biomass. Additionally, the study's kinetic analysis and optimization strategies provide valuable insights to enhance the sustainability and economic feasibility of bioethanol production.

## Materials and methods

### Sample collection and preparations

The lignocellulose feedstock used include (2 kg each) rice husk, maize straw, cob and husk, millet straw and husk, corn straw and husk, groundnut shell and sugarcane bagasse. Before characterisation, the samples were washed with distilled water and oven-dried for 48 h at 70 °C. The dried substrates were stored at room temperature in polyethylene bags.

### Lignocellulosic characterisation

The proximate analysis of the samples was conducted in accordance with ASTM Standard D7582-12. This included the determination of the moisture content, volatile matter, ash content, and fixed carbon. Moisture was measured by oven-drying at 105 °C until a constant weight was achieved. The volatile matter was determined by heating samples in a muffle furnace at 950 °C for 7 minutes, and the ash content was determined by combustion at 750 °C for 6 hours. Fixed carbon was calculated by difference.

The ultimate analysis was carried out based on ASTM Standard D3176-15, supplemented by established procedures from.^28^ The analysis quantified carbon (C), hydrogen (H), nitrogen (N), sulfur (S), and oxygen (O) content. A CHNS elemental analyzer (*e.g.*, PerkinElmer 2400 Series II) was used for C, H, N, and S, while the oxygen content was calculated by difference. All proximate and ultimate analyses were performed in duplicate, and the mean values were recorded. The standard deviation was calculated and reported in the corresponding results tables to reflect the experimental variability.

### Isolation of micro-organisms

*A. niger* was isolated from bread mold, and *T. reesei*, *Z. mobilis* and *S. cerevisiae* were isolated from decaying wood and termite nest. To produce a stock solution, the samples were combined with sterile distilled water (DW). Then, using sterile test tubes, a series of dilutions were made up to a dilution factor of 10^−7^. The microorganisms were cultured and maintained on Potato Dextrose Agar (PDA) from room temperature up to 32 °C, and cultured until a pure isolate was obtained as reported earlier.^29^

### Pretreatment of feedstock

Initially, two kg each of the feedstock was blended and delignified by ammonia steeping technique, as previously described by other authors.^30^ The feedstock was pretreated using diluted alkaline. A 250 ml Erlenmeyer flask containing 10 g of biomass and 100 ml of 2.9 M NH_4_OH was shaken for 24 h at 25 °C. Distilled water was used to filter and completely wash the feedstock. Additionally, a solid loading ratio was performed using a dry feedstock loading of 1 : 10 w/v biomass/water. The mixture was then autoclaved for one hour at 121 °C, allowed to cool to room temperature, and neutralised with 10 mol L^−1^ NaOH to reach pH 5.0. The pretreatment was conducted in duplicate for each feedstock type.

### Hydrolysis of feedstock

Samples were weighed in batches and transferred into a 100 ml shake-flask of 2 g each, and charged with 5 ml of media agar and inoculated with a suspension of 0.1 g of either *T. reesei* or *A. niger* with 100 ml of 0.1 M sodium acetate buffer solution at pH 4.5. The batches were then subjected to a shaker for agitation at 150 rpm and temperature of 50 °C. Furthermore, a separate batch of 2 g of treated and untreated feedstock was transferred to a 100 ml shaker flask, and 0.1 g of *T. reesei* or *A. niger* was added with 100 ml of 0.1 M sodium acetate buffer solution at pH 4.5. The flask was put on a shaker set at 50 °C and an agitation rate of 150 rpm, and allowed to equilibrate for 5 h. The samples were filtered and analyzed for glucose. Samples were collected every 0.5 h to examine the glucose concentration of the feedstocks using a spectrophotometric analyzer. The hydrolysis of both treated and untreated feedstocks was performed in duplicate shaker flask experiments under identical conditions.

### Determination of glucose

The dinitrosalicylic acid (DNS) colorimetric method was used to measure the amount of glucose present in the hydrolysed feedstocks, as described in the literature.^31^ It was assayed by adding 3 ml of 3,5-DNS reagents to 3 ml of the sample. The glucose concentration of the treated sample was determined for the feedstock with the highest cellulose. The effects of particle size and substrate pretreatment on the degree of hydrolysis were examined in a different run. Each run was duplicated, and the average value of each set of runs was reported.

### Effect of particle sizes of the feedstocks

The substrate of each feedstock was passed through different sizes of Tyler standard sieves. The particles sizes were <75 μm, 75 μm, 150 μm, 300 μm and 425 μm. Each particle size was hydrolyzed and the glucose concentration was analyzed.

### Fermentation

The 500 ml of filtrate that was obtained after the hydrolysis process (glucose syrup solution) was mixed with the fungus (*S. cerevisiae*) and bacteria (*Z. mobilis*) inoculum using the stationary method of fermentation. The reagent bottles that contained the glucose solutions were examined during the fermentation process to determine the amount of ethanol produced using a spectrophotometric method, as described earlier. The spectrophotometer was switched on and allowed to warm up for 10 min to stabilize. A standard ethanol calibration curve was first prepared by reacting known concentrations of ethanol with the chromogenic reagent. The reaction mixtures were incubated in a water bath at 60 °C for 20 minutes to ensure complete color development. A blank solution containing all reagents except ethanol was prepared and used to calibrate the spectrophotometer. The wavelength was set to the maximum absorbance for the ethanol–reagent complex, typically at 540 nm for the reagent used (potassium dichromate). Each sample was reacted with the same reagent under identical conditions. After incubation, the absorbance was measured against the blank. The ethanol concentration in the unknown samples was calculated by comparing their absorbance values with the standard calibration curve. All measurements were carried out in duplicate to ensure accuracy and reliability.

To support the growth of both *Z. mobilis* and *S. cerevisiae*, the hydrolysate's pH was brought to 5 prior to fermentation using sodium hydroxide (NaCl) and hydrochloric acid (HCl). To collects the distillate, the generated ethanol is poured into round-bottom flasks fastened to the opposite end of the distillation column. To heat the round-bottomed flask holding the ethanol–water mixture, the heating mantle's temperature was set to 78 °C.

### Monod equation

Microbial growth as a function of ethanol and total reducing sugar (TRS) concentration is related by the Monod kinetic expression, which includes the non-competitive substrate and product inhibition:^32^1
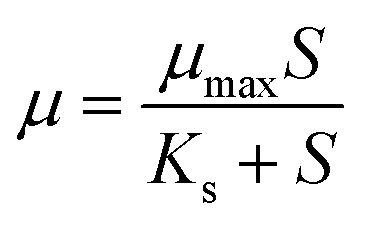
where, the limiting substrate concentration is *S* (g L^−1^), the maximum specific growth rate is *μ*_max_ (h^−1^), and the saturation constant is *K*_s_ (g L^−1^).

### Michaelis–Menten equation

Based on simplifications and presumptions like the quasi-steady-state assumption, the Michaelis–Menten rate law proposed an approximate kinetic formulation for the kinetic study of biological reactions. The kinetics of the enzymatic hydrolysis can be described by the Michaelis–Menten equation, as follows:^33^2
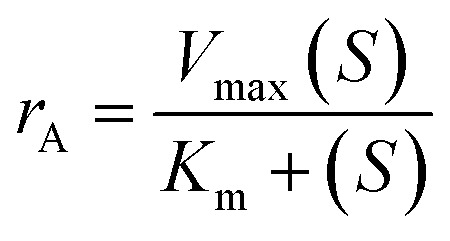
where *r*_s_ is the rate of reaction, *V*_max_ the maximum rate of the reaction, *S* the substrate concentration, and *K*_m_ is the Michaelis–Menten constant.3
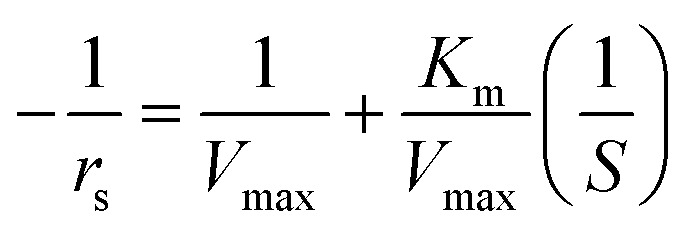


For a plot of 
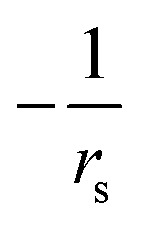
 against 
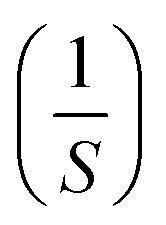
 for a straight line, *V*_max_ and *K*_m_ can be calculated from the intercept and the slope, respectively.

### Optimization procedure

Response Surface Methodology (RSM) was used to determine the optimal performance. Meanwhile, the central composite design (CCD), which developed the correlation between the independent variables (pH, substrate concentration, and fermentation time), was utilised to forecast and validate the model. The response is the dependent variable (ethanol production). The levels of factors are presented in Table 1. RSM analysis was performed based on duplicate experimental runs for each design point to ensure the accuracy and reliability of the predicted model. ANOVA was used to validate the model's statistical significance.

**Table 1:** Independent variables and their coded levels

Factors	Variables	Levels	
pH	*X*_1_	4.5	5
Substrate concentration	*X*_2_	1	5
Fermentation time	*X*_3_	35	50

Analysis of variance (ANOVA) was used to determine the statistical significance of the generated regression model. *P*-Values were estimated, with a *p*-value of less than 5% serving as the significance limit similar to previous studies in the literature.^34,35^

## Results and discussion

### Composition of the feedstocks

Proximate analysis was used to determine the moisture, fixed carbon, volatiles and ash content of the feedstocks. These levels are used to predict the behavior of the samples in hydrolysis and fermentation processes. The ash component of the proximate analysis defines the inorganic residue remaining after ignition of the combustible biomass, while the fixed carbon and volatile matter content significantly affect the presence of the C, H, and O content of biomass.^36,37^

For bioethanol production, feedstocks with high volatile matter, moderate moisture content, and low ash content are preferred, as they contain more fermentable carbohydrates.^11^ The proximate results are presented in Table 2.

**Table 2:** Proximate analysis of biomass feedstocks with estimated error bars

Feedstock	Ash (%)	Moisture content (%)	Fixed carbon (%)	Volatile matter (%)
Millet husk	9.95 ± 0.12	4.56 ± 0.10	5.76 ± 0.15	40.2 ± 0.5
Millet cob	11.79 ± 0.15	2.78 ± 0.09	37.34 ± 0.40	42.6 ± 0.6
Sorghum husk	11.50 ± 0.14	3.14 ± 0.10	41.49 ± 0.45	44.7 ± 0.5
Sorghum cob	12.00 ± 0.15	4.23 ± 0.11	38.39 ± 0.40	56.2 ± 0.7
Maize husk	11.22 ± 0.13	4.76 ± 0.12	38.97 ± 0.35	33.2 ± 0.6
Maize cob	13.00 ± 0.15	4.11 ± 0.11	2.58 ± 0.10	31.1 ± 0.4
Maize straw	10.50 ± 0.12	4.42 ± 0.10	2.58 ± 0.10	33.5 ± 0.5
Rice husk	10.37 ± 0.13	2.11 ± 0.08	38.61 ± 0.38	62.2 ± 0.8
Groundnut shell	15.37 ± 0.18	4.63 ± 0.12	31.50 ± 0.33	34.0 ± 0.5
Sugarcane bagasse	10.97 ± 0.14	2.74 ± 0.09	11.60 ± 0.25	63.4 ± 0.8

The proximate and ultimate analysis results are presented as mean ± standard deviation, based on two replicates. For example, the ash content of millet husk was 9.95% ± 0.17%, and the carbon content of rice husk was 47.2% ± 0.46%. This accounts for variability in the sample handling and equipment precision.

Sorghum cob, millet cob, and rice husk are ideal due to their high volatile matter and relatively low moisture and ash content, making them rich in cellulose and hemicellulose for enzymatic hydrolysis. Sugarcane bagasse is also a strong candidate due to its widespread use in ethanol production. The lower fixed carbon, as seen in sugarcane bagasse (11.60%), indicates lower lignin content, reducing the need for extensive pre-treatment. However, feedstocks with high ash content may pose challenges due to the non-fermentable residues.^38,39^ Overall, sorghum cob, millet cob, rice husk and sugarcane bagasse are the most suitable for bioethanol production based on their composition.^40^

The final analysis shows how much cross-linking there is, and how many high molecular weight compounds there are in the feedstocks. The final examination of feedstocks aids in determining their eligibility for the manufacturing of bioethanol based on the concentration of carbon (C), hydrogen (H), oxygen (O), nitrogen (N), and sulphur (S) (Table 3).^41^

**Table 3:** Ultimate analysis of feedstocks with ± SD

Feedstock	Carbon	Hydrogen	Nitrogen	Oxygen	Sulphur
Millet husk	42.40 ± 4.24	6.32 ± 0.63	0.03 ± 0.00	61.00 ± 6.10	0.13 ± 0.01
Millet cob	41.80 ± 4.18	6.36 ± 0.64	0.02 ± 0.00	94.00 ± 9.40	0.82 ± 0.08
Sorghum husk	43.70 ± 4.37	7.41 ± 0.74	0.02 ± 0.00	69.00 ± 6.90	0.51 ± 0.05
Sorghum cob	43.10 ± 4.31	7.70 ± 0.77	0.03 ± 0.00	47.00 ± 4.70	0.32 ± 0.03
Maize husk	44.10 ± 4.41	7.86 ± 0.79	0.02 ± 0.00	62.00 ± 6.20	0.11 ± 0.01
Maize cob	42.90 ± 4.29	6.99 ± 0.70	0.02 ± 0.00	71.00 ± 7.10	0.20 ± 0.02
Maize straw	43.10 ± 4.31	6.99 ± 0.70	0.02 ± 0.00	71.00 ± 7.10	0.20 ± 0.02
Rice husk	47.20 ± 4.72	7.27 ± 0.73	0.02 ± 0.00	57.00 ± 5.70	0.11 ± 0.01
Groundnut shell	41.80 ± 4.18	7.32 ± 0.73	0.08 ± 0.01	20.00 ± 2.00	0.29 ± 0.03
Sugarcane bagasse	49.80 ± 4.98	8.44 ± 0.84	0.02 ± 0.00	67.00 ± 6.70	0.05 ± 0.01

The high carbon and hydrogen content indicates the presence of carbohydrates necessary for fermentation, while the oxygen content reflects the proportion of cellulose, hemicellulose, and lignin. Sugarcane bagasse (49.8% C, 8.32% H) and rice husk (47.2% C, 8.27% H) have the highest carbon content, suggesting the presence of a significant amount of lignocellulosic material. However, they may require pre-treatment to break down the lignin and release the fermentable sugars. Sugarcane bagasse^42^ and rice husk^38^ are considered as good candidates, which is partially due to their high oxygen content, indicating a good proportion of carbohydrates. Low nitrogen and sulfur levels are desirable, as they reduce the formation of inhibitory compounds during fermentation. Sugarcane bagasse (0.018% N, 0.05% S) and rice husk (0.019% N, 0.11% S) have the lowest levels, making them favorable for ethanol production.

### Glucose composition

**Fig. 1:**
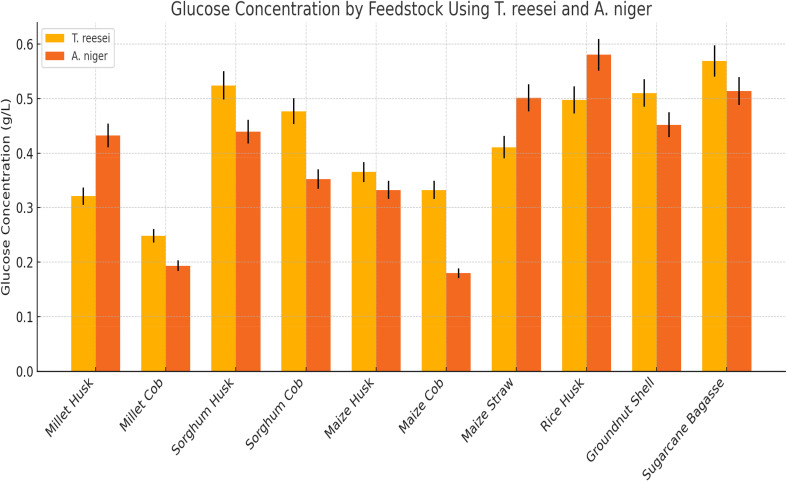
Glucose concentration.

The cellulose component in Sugar bagasse (SB) with respect to *A. niger* hydrolysate was found to be the highest compared to the other nine feedstocks in this study. Meanwhile, the lowest was found in millet cob. Furthermore, the cellulose component in Rice Husk (RH) with respect to *T. reesei* hydrolysate was found to be the highest with the least from maize cob. Fig. 1 shows the glucose concentration of the feedstocks after 10 h.

The glucose concentrations of 0.5689 and 0.5803 g L^−1^ were found for the sugarcane bagasse with *T. reesei* (SBTr) and rice husk with *A. niger* (RHAn), respectively. Hence, these two feedstocks were used for further study. This study demonstrated a low hydrolysis time of 10 h due to the nature of the feedstock. Additionally, cheap production costs for industrial use and a fast hydrolysis time would be advantageous for small production processes.^43–46^

### Effect of pretreatment on feedstocks

**Fig. 2:**
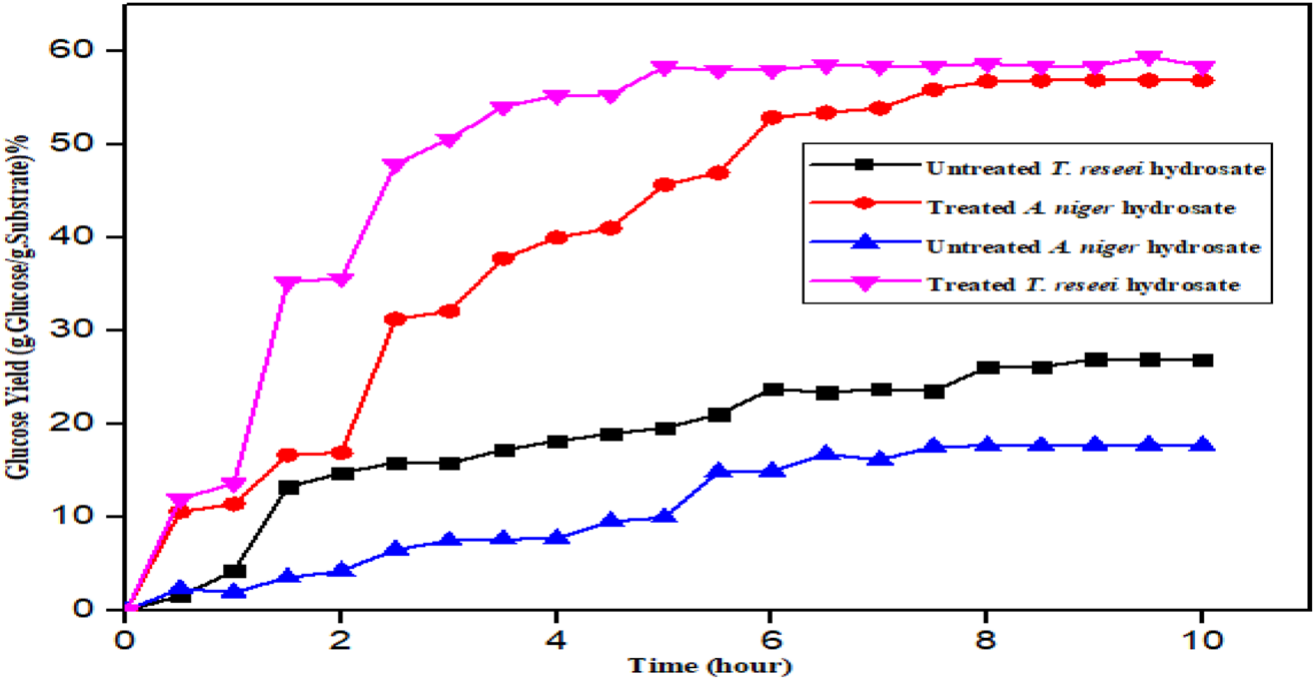
Treated and untreated glucose yields of SBTr and RHAn.

The effects of pretreatment on the feedstocks and the glucose concentration with respect to time are presented in Fig. 2 for treated and untreated glucose concentration of SBTr and RHAn.^47^

Polysaccharide is typically left behind after alkaline pretreatment techniques remove the lignin component from lignocellulosic biomass. Meanwhile, acid pretreatment techniques have a higher recovery of cellulose components because the hemicelluloses are removed.^48,49^ The untreated samples yield less glucose concentration compared to the treated substrate due to the absences of lignin and hemicelluloses or their inhibition (lignin obstructs hydrolysis). The pretreatment increases the substrate's inner surface area, which helps to create an environment that is favorable for enzymatic hydrolysis to occur. The glucose yield from RHAn is greater than that of SBTr. This is because the three major factors in enzymatic hydrolysis are the nature of the enzyme, the structure of the substrate system, and the interactions between the enzyme and substrate. This finding shows that pretreatment in enzymatic hydrolysis is beneficial in cellulose production, which agrees with other studies.^50–52^

### Effects of particle size on the feedstocks

**Fig. 3:**
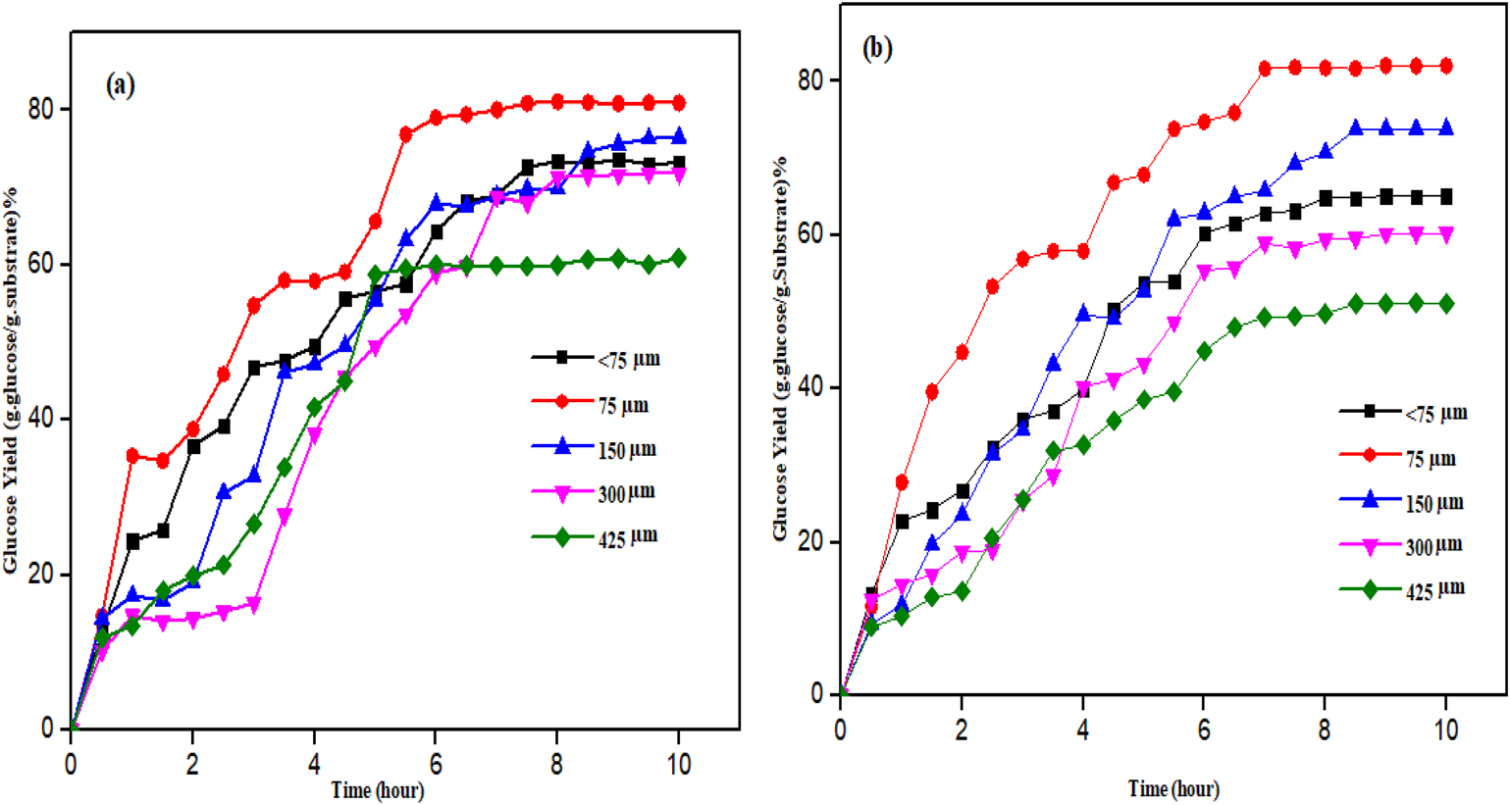
Effects of particle size on glucose concentration in (a) SBTr and (b) RHAn.

A key factor in the economy of glucose production is the feedstock particle size, which typically has a beneficial impact on the hydrolysis glucose yields. Fig. 3 shows the effects of the particle size on the glucose concentration.

In this study, it was found that the improved yield can be ascribed to the increase in the specific area and the cellulose availability for hydrolysis. By increasing the specific surface area, the decreasing particle size improves the hydrolysis yields. The best-performing particle size was 75 μm for the glucose concentration in both SBTr and RHAn. Aderemi *et al.* (2008) observed that when the size of the rice straw particles decreased from 425 to 75 μm, the amount of glucose produced increased from 43% to 87%. However, further reduction of the particle size below this point has no effect on the amount of glucose produced.^30^

### Kinetic model for hydrolysis

**Fig. 4:**
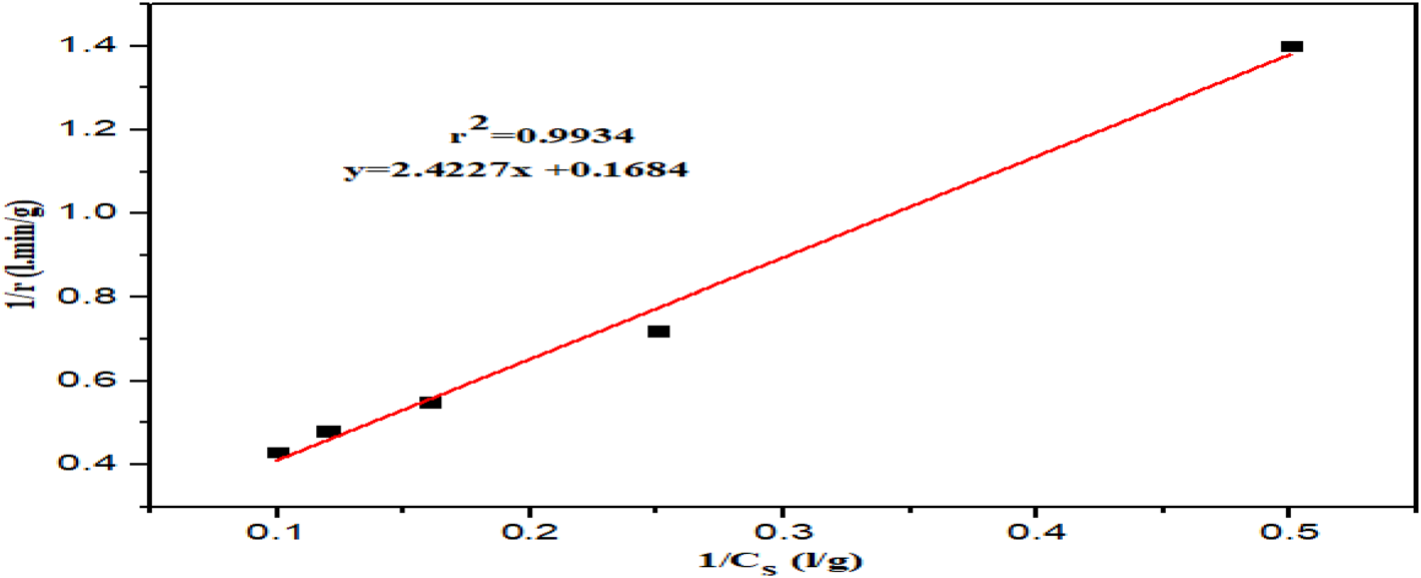
Lineweaver–Burk plot of Michaelis–Menten kinetics.

The Lineweaver–Burk linearization models of the Michaelis–Menten equation was used to determine the *r*_max_ and *K*_m_ values. Fig. 4 depicts the Lineweaver–Burk plot of the Michaelis–Menten kinetics.

The kinetic parameters were determined using the Lineweaver–Burk model at a fixed temperature (50 °C), pH (5) and enzyme amount (0.1 g L^−1^) with varied substrate concentration. The kinetic parameters were estimated, where *r*_max_ is 5.9 g L^−1^ min^−1^ and *K*_m_ is 14.41 g L^−1^, respectively. The slope reflects the enzyme efficiency, helping to optimize the bioethanol production by identifying the ideal feedstock and enzymatic conditions. Previously, *A. niger* was used to hydrolyze the sago starch, where the kinetic parameters *r*_max_ and *K*_m_ were determined to be 4.78 g L^−1^ min^−1^ and 0.6 g L^−1^, respectively.^53^ The substrate plays a key role in determining the kinetic parameters. The disparity in the obtained values are due to the differences in the hydrolysis conditions, including the use of different substrates. A similar scenario has been reported earlier.^54^ According to the evaluated kinetic parameters, the model equation in this study is given as follows:4
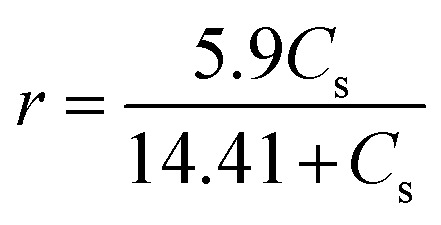


**Fig. 5:**
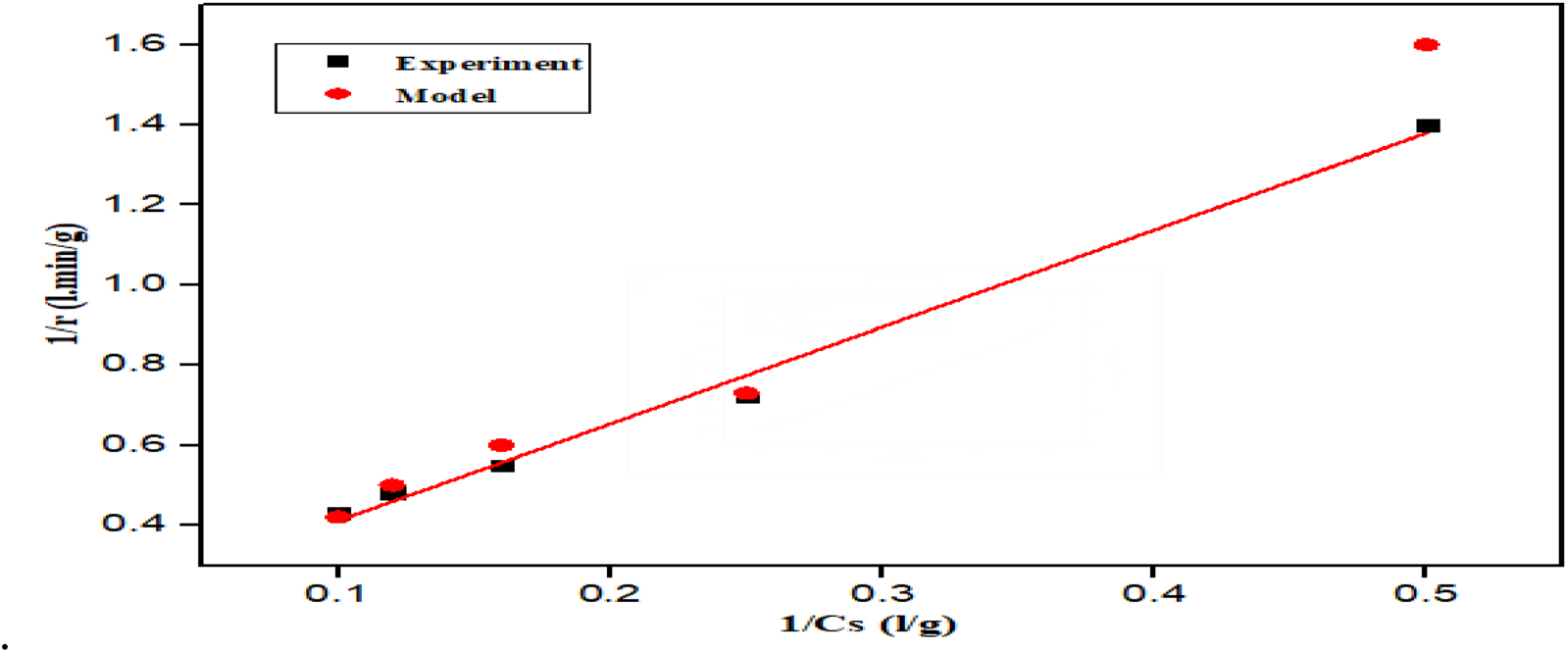
Comparison of model predicted rate with experimental results.

The consistency of this model equation was tested with the generated data to evaluate its reliability. A comparison of the model-predicted rate against the experimentally obtained rate is shown in Fig. 5.

To validate the model, a comparison between the predicted and experimentally observed hydrolysis rates was performed, as shown in Fig. 5. To further bolster the reliability of the kinetic model, the following statistical analyses were conducted. The *R*^2^ value for the model fit was 0.981, indicating that the kinetic model explained 98.1% of the variability in the experimental data. The Root Mean Square Error (RMSE) between the predicted and observed rates was 0.23 g L^−1^ min^−1^, confirming the low predictive error. Residual plots showed no clear trend, suggesting that the model assumptions (linearity and homoscedasticity) were valid and the residuals were randomly distributed.

These metrics confirm that the Lineweaver–Burk model provided an excellent fit to the experimental data, supporting its use for predictive analysis in optimizing the hydrolysis conditions. The validated kinetic model can be used for scale-up simulations and optimizing operational parameters for enzymatic hydrolysis in bioethanol production.

### Fermentation of hydrolysates

Alkali/acid pretreatment was carried out as necessary before fermentation, and the hydrolysates that had been pretreated were then fermented. Hydrolysate from the sugar bagasse and rice husk have shown the highest cellulose generation based on the *A. niger* and *T. reesei* strains. Therefore, they were selected for fermentation using mixed *S. cerevisiae*/*Z. mobilis*, with *S. cerevisiae* and *Z. mobilis* as the inoculums. Furthermore, a mixed culture of *S. cerevisiae*/*Z. mobilis* was studied since only limited research has examined how the two microbes interact to affect the hydrolysate made from lignocellulosic materials.

**Fig. 6:**
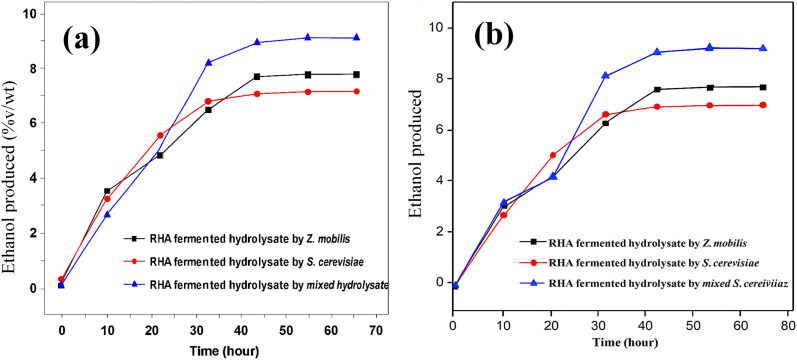
Ethanol production over fermentation time: (a) SBTr fermented hydrolysate and (b) RHAn fermented hydrolysate.

Under the fermentation conditions of 30 °C, pH 5 and 120 rpm, the reducing sugar consumption indicated the existence of a nutrient limitation or inhibitory metabolites in the medium, while the accumulation of reducing sugar represses the production of cellulose.^55^Fig. 6 shows the ethanol production with fermentation time.

**Fig. 7:**
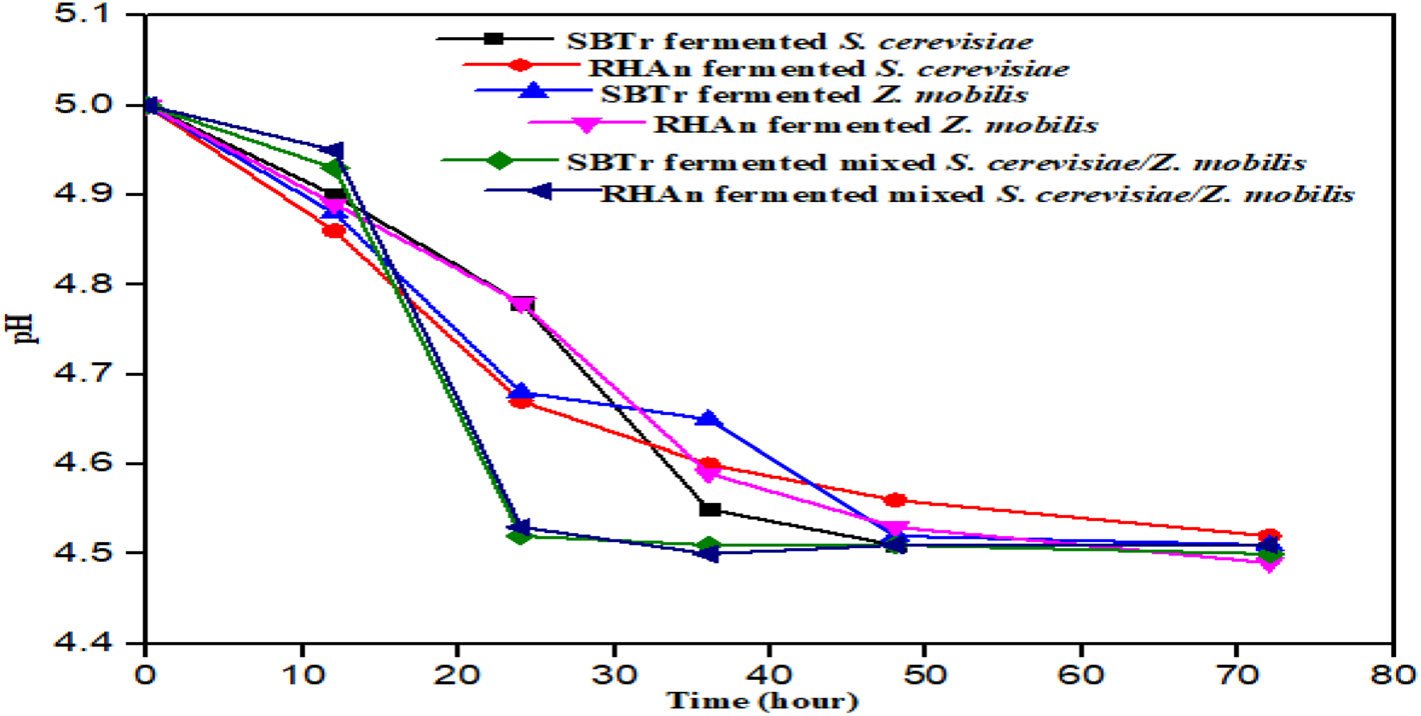
Change in pH for various hydrolysates.

The fermentation study demonstrated that the mixed *S. cerevisiae*/*Z. mobilis* cultures produced the highest ethanol yield in both SBTr and RHAn hydrolysates, indicating a synergistic effect between the two strains. The peak ethanol production times varied, with mixed cultures reaching their highest yield at 60 h for RHAn hydrolysate (9.3 g L^−1^) and 48 h for SBTr hydrolysate (8.1 g L^−1^), surpassing the individual strains. However, the combination of both strains likely enhances sugar utilization, overcoming the challenge of fermenting hexose and pentose sugars in lignocellulosic hydrolysates. This highlights the potential of mixed cultures to improve ethanol yield and reduce production costs, making bioethanol production more viable. This finding aligned with the literature finding.^23^ Additionally, factors like pH, temperature, and incubation time play crucial roles in optimizing microbial metabolism, emphasizing the need for precise control of fermentation conditions for maximum ethanol output.^56^Fig. 7 shows the change in pH for various hydrolysates during fermentation.

The gradual decrease in pH from 5 to around 4.5 during fermentation indicates microbial activity and the production of organic acids alongside ethanol synthesis. The stabilization of pH at 4.5 aligns with the optimal range for yeast and bacterial fermentation, ensuring efficient enzymatic activity. The observation that ethanol production is influenced by pH and incubation temperature supports previous studies,^57,58^ emphasizing the need for controlled fermentation conditions to maximize yield. The ability of the tested strains to adapt to the fermentation environment suggests their suitability for large-scale ethanol production. Given the importance of pH in microbial metabolism, further investigations into the optimal pH range (4.5–4.7) could enhance the ethanol yield, making the process more efficient and economically viable.

### Kinetic model for ethanol production

**Fig. 8:**
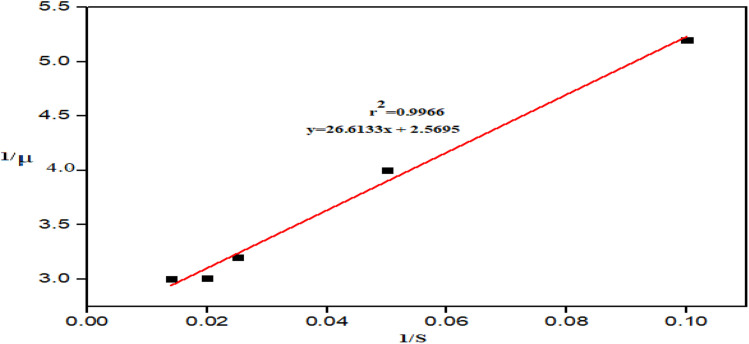
Plot of the reciprocals of specific microbial growth rates against the reciprocals of substrate concentration.

The ethanol produced with RHAn hydrolysate demonstrated the highest yield with the mixed *S. cerevisiae*/*Z. mobilis* of 9.3 g L^−1^. It is therefore used for the study of kinetic growth using the Monod equation. Fig. 8 shows the plot of reciprocals of specific microbial growth rates against reciprocals of the substrate concentration.

The kinetic parameters *μ*_max_ (2.5695) and *k*_s_ (26.6133) exhibited a very high regression coefficient *r*^2^ = 9966, as determined from the intercept and slope, respectively. The *r*^2^ = 0.9966 value indicates that the model adequately describes the production of ethanol by the mixed *S. cerevisiae*/*Z. mobilis* culture during fermentation of RHAn hydrolysate. Therefore, the model equation is as follows:5
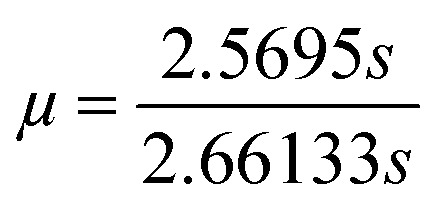


Other researchers^59^ reported on the growth kinetic parameters for rice hydrolysate fermented by *C. acetobutylicum* as *μ*_max_ (4.4649) and *k*_s_ (2.8035) with *r*^2^ = 0.9823. The same researchers stated that the lower *k*_s_ (<5) value shows the innate affinity of the microorganism for the substrate because its reciprocal characterises the cell's affinity for the substrate.

### Ethanol production optimization

Table 4 presents the experimental design for ethanol production using a CCD, where the pH (A), substrate concentration (B), and fermentation time (C) were varied across 20 experimental runs to observe their effects on the ethanol yield (%V/Wt).

**Table 4:** Experimental design based on CCD for ethanol production

Run	*A*: pH	*B*: Substrate concentration (g L^−1^)	*C*: Fermentation time (Hour)	Ethanol production (%V/Wt)
1	5.00	5	35.0	4.80
2	4.75	1	42.5	7.50
3	4.5.0	1	35.0	9.70
4	4.75	5	42.5	5.30
5	4.75	3	42.5	6.90
6	4.75	3	35.0	6.70
7	5.00	1	35.0	6.70
8	5.00	1	50.0	8.40
9	4.50	1	50.0	10.5
10	4.50	5	50.0	8.60
11	4.75	3	42.5	6.70
12	4.75	3	42.5	7.00
13	5.00	3	42.5	5.70
14	4.75	3	42.5	7.10
15	4.75	3	50.0	8.30
16	4.75	3	42.5	7.20
17	4.75	3	42.5	7.00
18	5.00	5	50.0	6.30
19	4.50	5	35.0	7.40
20	4.50	3	42.5	8.40

The results indicate that ethanol production fluctuates with changes in these factors, with the highest yield (10.5% V/Wt) occurring at pH 4.5, substrate concentration of 1 g L^−1^, and fermentation time of 50 hours (Run 9). In contrast, lower ethanol yields were observed at higher pH values (*e.g.*, Run 1 with pH 5.0, yielding 4.8%). This suggests that a lower pH (around 4.5) and longer fermentation time enhance ethanol production, aligning with optimal microbial fermentation conditions. This study highlights the significance of optimizing these parameters to efficiently maximize the ethanol yield.

Table 5 presents the Analysis of Variance (ANOVA) results for ethanol production, evaluating the statistical significance of the factors (pH, substrate concentration, and fermentation time) on the ethanol yield.

**Table 5:** Analysis of variance (ANOVA) for ethanol production

Source	Sum of squares	df	Mean square	*F* value	*p*-Value Prob > *F*	
Model	35.89	9	3.99	73.13	<0.0001	Significant
*A*-pH	16.13	1	16.13	295.75	<0.0001	
*B*-substrate concentration	10.82	1	10.82	198.33	<0.0001	
*C*-Fermentation time	4.62	1	4.62	84.79	<0.0001	
*AB*	5.000 × 10^−3^	1	5.000 × 10^−3^	0.092	0.7683	
*AC*	0.18	1	0.18	3.30	0.0993	
*BC*	5.000 × 10^−3^	1	5.000 × 10^−3^	0.092	0.7683	
*A* ^2^	0.36	1	0.36	6.67	0.0273	
*B* ^2^	0.23	1	0.23	4.14	0.0694	
*C* ^2^	1.82	1	1.82	33.38	0.0002	
Residual	0.55	10	0.055			
Lack of fit	0.40	5	0.079	2.68	0.1519	Not significant
Pure error	0.15	5	0.030			
Cor total	36.44	19				

The model is significant (*p* < 0.0001, *F* = 73.13), indicating that the chosen factors strongly influence ethanol production. Among the individual factors, pH (*A*) has the highest impact (*F* = 295.75, *p* < 0.0001), followed by the substrate concentration (*B*, *F* = 198.33, *p* < 0.0001) and fermentation time (*C*, *F* = 84.79, *p* < 0.0001). The quadratic terms *A*^2^ (*p* = 0.0273) and *C*^2^ (*p* = 0.0002) also significantly affect the ethanol yield, suggesting a nonlinear relationship. However, the interaction terms *AB*, *AC*, and *BC* are not significant, indicating that the combined effects of these factors do not notably impact ethanol production. The lack of fit is not significant (*p* = 0.1519), confirming the model's reliability for predicting the ethanol yield. The developed empirical model equation represents the relationship between the ethanol production (response variable) and the independent factors:

6 Ethanol production (g L^−1^) = 6.86 − 1.27 × *A* − 1.04 × *B* + 0.68 × *C* + 0.025 × *AB* + 0.15 × *AC* + 0.025 × *BC* + 0.36 × *A*^2^ − 0.29 × *B*^2^ + 0.81 × *C*^2^

This equation indicates that the pH and substrate concentration have a negative effect on the ethanol yield, meaning that higher values reduce the ethanol production. The fermentation time has a positive effect, suggesting that a longer fermentation time enhances the ethanol yield. The interaction terms *AB*, *AC*, and *BC* are minor, indicating weak combined effects. This model helps predict the ethanol yield based on the selected fermentation conditions, and guides the optimization for the maximum production efficiency.

### Response surface analysis

**Fig. 9:**
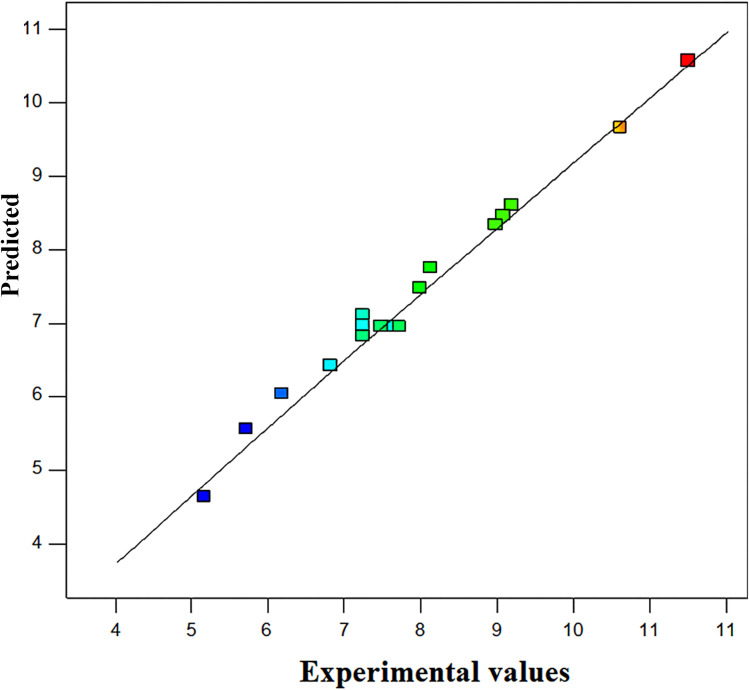
Predicted values *versus* the actual values for the ethanol production.

As shown in Fig. 9, the predicted and actual values are quite similar. This graphic suggests that independent variables and the response can be correlated by the developed model. This pattern suggests that the model is appropriate for the current investigation.

**Fig. 10:**
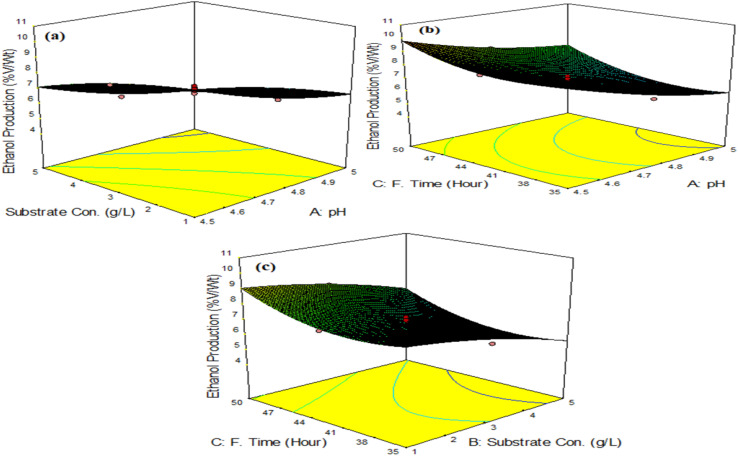
Surface plots of interactions as a function of (a) substrate concentration and pH, (b) fermentation time and pH, and (c) fermentation time and substrate concentration.

Plotting the model's surface responses clearly demonstrates how the independent variables affect the responses. Fig. 10 shows the response surface plots, displaying the ethanol production across various independent variable combinations and their combined effects.

Fig. 9 presents the interactive effects of ethanol production with two independent variables, while keeping the other variable at a fixed level. At high and low levels of some of the interactions, the ethanol production is minimal. However, there is a region where no change occurred in the adsorption capacity. This region shows that an optimum ethanol production for the variables exists. It can be deduced from Fig. 10(a) (due to the lack of a definite curvature) that the ethanol production is not appreciable as a result of the influence of the substrate concentration and pH (6.6 g L^−1^) when compared to the influences of the fermentation time and pH (9.4 g L^−1^), as depicted in Fig. 10(b). The curved contour lines reveal that there is an interaction between the fermentation time and pH, whose combined effect influences the ethanol production. Furthermore, the fermentation time and substrate concentration in Fig. 10(c) demonstrate less influence on the ethanol production (8.6 g L^−1^) when compared to Fig. 10(b).

When *P* < 0.001, the interaction between the independent variables is highly significant. The results of the analysis of variance and the regression model's test for significance match the performances represented in the curved contour lines. These findings show that the model provides a sufficient explanation of the experimental range under study. The fitted regression equation indicates the association between the independent variables, and demonstrates a satisfactory fit of the models. There are reports of similar studies.^11,57^

The optimization analysis from the software gave the results as selected based on the response results fed to the software. The comparison between the predicted ethanol production (10.5 g L^−1^) and the experimental result (10.1 g L^−1^) shows a small difference of 0.4 g L^−1^, indicating a high level of accuracy in the developed model. Since this variation is minimal, the model can be considered a good fit for predicting the ethanol production under the given conditions. This validates the reliability of the empirical equation in estimating the ethanol yield, and suggests that the model can be used to optimize the fermentation parameters for improved efficiency.^60^

## Conclusion

This study evaluated the suitability of various lignocellulosic feedstocks for bioethanol production based on their proximate and ultimate compositions, glucose yield, and fermentation efficiency. Sorghum cob, millet cob, rice husk and sugarcane bagasse demonstrated optimal volatile matter, moisture, and ash content, making them promising candidates. Ultimate analysis revealed that rice husk and sugarcane bagasse exhibited high oxygen and hydrogen content, enhancing the enzymatic hydrolysis efficiency. Among the tested feedstocks, sugarcane bagasse and rice husk produced the highest glucose yields when hydrolyzed using *A. niger* and *T. reesei*, respectively, with alkaline and acid pretreatments significantly improving the conversion rates. Fermentation studies confirmed that mixed cultures of *S. cerevisiae* and *Z. mobilis* achieved the highest ethanol yields, demonstrating synergistic sugar utilization. These findings highlight the potential of optimized ethanol production and microbial selection to enhance bioethanol production. Furthermore, it shows the kinetics models for hydrolysis and fermentation processes. Although the specific quantification of cellulose, hemicellulose, and lignin was not conducted in this study, future work will incorporate these analyses to refine the kinetic modeling and substrate-microbe compatibility, particularly for mixed microbial systems targeting specific saccharides.

## Conflicts of interest

The authors declare no conflict of interest.

## Data Availability

All data will be made available from the corresponding authors upon reasonable request.
